# Socio-Emotional Competencies Required by School Counsellors to Manage Disruptive Behaviours in Secondary Schools

**DOI:** 10.3390/children10020231

**Published:** 2023-01-28

**Authors:** Ángela Serrano, Roberto Sanz, Juan Luis Cabanillas, Elena López-Lujan

**Affiliations:** 1 Faculty of Teaching and Educational Sciences, Department of Inclusive Education, Socio-Community Development and Occupational Sciences, Catholic University of Valencia, Godella, 46110 Valencia, Spain; 2 Facultad de Magisterio y Ciencias de la Educación, Departamento de Didáctica General, Teoría de la Educación e Innovación Tecnológica, Catholic University of Valencia, Godella, 46110 Valencia, Spain; 3 Facultad de Educación, Universidad de Salamanca, 37008 Salamanca, Spain

**Keywords:** social-emotional competencies, school counsellors, conflict, mental health, conflict resolution

## Abstract

This article identifies the socio-emotional competencies of school counsellors working with children and adolescents. The aim is to address problems related to mental health and conflict and to implement training programmes. The study sample was composed of 149 counsellors working in schools. The instruments used were the CCPES-II (questionnaire on teacher competences) and a series of open-ended questions on conflict resolution. A mixed methodology was used, with a concurrent triangulation design with two phases: a quantitative one (QUAN) and a qualitative one (QUAL). Univariate, bivariate, and correlation quantitative analyses were performed. Parametric and non-parametric tests were applied depending on the number of dependent and independent variables. The qualitative analysis was performed with the NVivo 12 computer programme, which determines word frequencies using a classic content analysis. The results confirm the relationship between socio-emotional training and rapid response to school conflict; the generalised view that conflicts are difficult to anticipate and, thus, to prevent; and the demand for specific training in socio-emotional competences, intervention strategies, more specialised school staff, more time for intervention with and support for families, and more socio-professional recognition.

## 1. Introduction

Multiple studies have shown that, after the COVID-19 pandemic, mental-health problems in children and adolescents have increased and that the social-care system has been unable to adequately tackle this problem [1,2,3,4,5]. For example, in a systematic review of 53 studies, Radez et al. [6] identified the difficulties in responding to mental health-related conflict situations. Of the studies reviewed, 58% found structural problems in healthcare systems. These problems are related to the financial cost associated with mental-health care as well as logistical barriers and the availability of professional help. These problems are exacerbated among children and adolescents because the educational system often lacks professionals trained in mental-health and conduct problems, with school counsellors having to deal with these issues [7]. 

Also striking is the large amount of school conflicts handled by educational guidance services that show the relationship between the early onset of untreated personal conflicts and later serious conduct problems entailing possible victimisation [8], suicide, or bullying.

In order to provide an adequate response to these problems, training programmes for school counsellors should use a combined psychological, pedagogical, and educational approach that features not only their traditional subjects but also certain socio-emotional competencies. Unfortunately, in some cases, training programmes do not cover these needs. 

The aim of this article is to identify the socioemotional competencies required by school counselors to respond to the different mental health problems, conflicts, and behavioural problems presented by students in secondary education (12–16 years old) in schools, as well as to detect their needs and analyse the main strategies and educational measures of action that they implement in their professional work. This article aims to respond to a need detected in previous research among secondary education teachers.

### 1.1. Mental-Health Problems in Children and Adolescents

Various studies have revealed the impact of COVID-19 on children and adolescents. Problems such as anxiety, emotional irritability, guilt, pain, despair, and fear have all been observed [9,10,11,12,13,14], and the physical restrictions and social distancing have affected every area of their lives. While it is difficult to determine exactly how many children and adolescents have been affected, it is clear that containment and social-distancing measures, isolation, and school closures are having a serious impact on their mental health and social wellbeing [11]. One thing the pandemic has exposed is the problem of mental health in children, a problem that has been ignored for a long time [15]. 

UNICEF [15] provides worrying data on mental health in children and adolescents: suicide is the fifth-leading cause of death among adolescents aged 10 to 19, preceded by traffic accidents and substance abuse, with interpersonal violence coming in third. Several studies have concluded that depression increased significantly among small children and that addiction to new technologies is currently one of the least known and least treated problems.

When faced with painful situations, small children usually react with anger and anguish, which subsequently turn into behavioural problems. In contrast, adolescents tend to react to emotional pain with sensation-seeking, self-harm, eating disorders, addictions, and alcohol/drug abuse. The studies conducted on the consequences of the pandemic have made it possible to determine the mental-health situation in the population, especially in children and adolescents. The results for the 10 to 19 age group are especially alarming and show that the pandemic has exposed a social problem that had been ignored for a long time [16,17,18]. This is true in different parts of the world [12,14,19,20,21,22,23]. Therefore, profound reflection is needed in the educational field because children and adolescents spend a lot of time in school, and this environment should be a protective factor against mental-health problems such as social maladjustment, depression, bipolarity, feelings of insecurity, suicidal tendencies, the risk of victimisation in school, conduct problems, and the use of violent behaviours by some students [24], among others.

### 1.2. School Conflict: The Current Situation

Different types of conflicts take place in schools, such as intrapersonal conflicts (identity or self-esteem problems, etc.), interpersonal conflicts (disruption, indiscipline, behaviour problems such as violence, and situations springing from functional diversity, such as autism or intellectual disability, etc.), and finally, conduct disorders. All of them are linked to a series of risk factors, a term defined as those variables that place minors in situations of vulnerability.

In spite of the many studies that have been conducted internationally on school relations and conflicts, it is hard to find clear and homogeneous statistics showing the state of the question. This is often because different studies use different methods and study different things. What is clear across all studies, however, is that there exists a wide range of conflicts in schools depending on the type of relationship, whether vertical—between parents and teachers, between teachers and students, etc.—or horizontal—among teachers, among students, etc. [25].

Regarding conflicts in general, studies show that one in four students has witnessed some type of school conflict, be it a simple disruption or more complex problems such as conduct disorders or violence. The latest OCDE report [26] states that 14% of schools have at least one conflict caused by student behaviour per week and 25% of students experience bullying or cyberbullying [27,28,29,30]. The other types of conflict students face are related to indiscipline, demotivation, apathy, failure to adapt to the peer group, lack of emotional control, and impulsivity. All of these negatively impact classroom relations [29,31,32], creating a negative atmosphere and causing some degree of emotional exhaustion in those tasked with managing the classroom. Furthermore, some studies point out that teachers and counsellors do not apply (and do not know) [33] any preventive strategies for conflict resolution. In fact, the strategies that are used are reactive and punitive in nature. Preventive strategies are those that promote the negotiation of rules, the establishment of early positive relations (before conflicts emerge), school mediation, and conflict arbitration, while punitive strategies are related to penalties and punishments.

#### Competences of Educational Guidance Counsellors for Conflicts: Challenges and Perspectives

Normally in Europe and specifically in Spain, emotional and behavioural conflicts of students in schools are dealt with by guidance counsellors (pedagogues or psychologists), who are presumed to possess the abilities necessary to manage such conflicts. However, dealing with these conflicts requires prior training in various competences because counsellors may have to deal with all manner of situations, and this requires specific training [34], which they have not always received. Among the functions performed by school counselors in schools are detecting, diagnosing, and intervening in social and behavioural problems and specific educational needs for diversity. For mental health problems, such as behavioural disorders, the counsellor refers to specialists in the health field (a psychiatrist and a clinical psychologist).

The university training curriculum for guidance counsellors covers a wide range of disciplines but has no specific subject that consolidates the socio-emotional skills needed for successful conflict resolution [35]. In most cases, this training consists of complementary courses that are mostly informative and do not include any follow-up process [36].

Several studies indicate that training a guidance counsellor to be highly competent in conflict resolution requires combining resources, specific knowledge, and interpersonal and intrapersonal competences because one needs to manage not only other people’s emotions but also one’s own [35,37,38]. Intrapersonal emotional competences are related to emotional self-knowledge, self-regulation, and motivation. Interpersonal competencies, on the other hand, are related to social skills, empathy, assertiveness, active listening, and communication strategies. Therefore, when teachers and guidance counsellors establish positive relations with their students, it protects them from risks such as alcohol and drug abuse, gang membership, self-harm, and suicide, in addition to generating a positive emotional climate that prevents the emergence of conflicts [33].

## 2. Materials and Methods

This study used a mixed method with a concurrent triangulation design that had two phases: phase 1, which was quantitative (QUAN), and phase 2, which was qualitative (QUAL). Both phases collected data simultaneously and separately. The analysis of one set of data was not based on the analysis of the other because the results of both types of analysis were not consolidated in each method’s interpretation phase but after both data sets had been collected and analysed separately. Once both types of data had been collected and their results analysed, one or more comparisons were performed that integrated both methods’ findings and conclusions and their connection [39,40].

**Figure 1:**
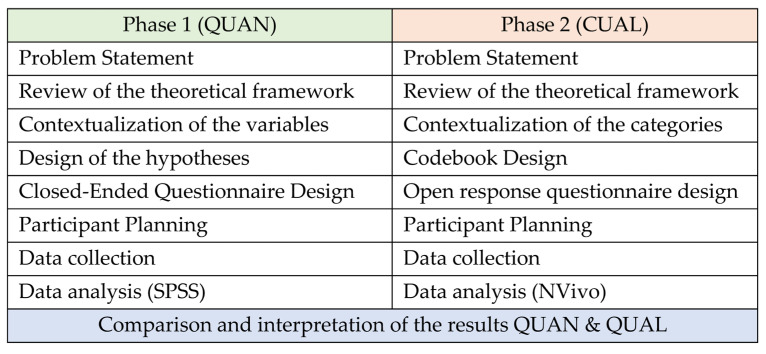
Outline of investigation procedures.

Before initiating the three research phases, a theoretical framework was developed to clarify the concepts to be worked on in the research. Likewise, the research objectives were established. Figure 1 shows the methodological procedures followed in this research, which consisted of three phases:

Phase 1. Methodological preparation. Definition of variables and analytical categories, instrument design, and sampling method.

Phase 2. Data collection and analysis. Data are collected through the questionnaire sent to the 149 counsellors and pedagogues participating in the training program.

Phase 3. Results. Data analysis, comparison, and interpretation of the results. These results are presented quantitatively (QUAN) and qualitatively (QUAL), respectively.

For the QUAN analysis, the programme SPSS (IBM 25) was used. The following statistical analyses were performed: (a) univariate for the description of the general characteristics of the sample; (b) bivariate to observe the differences between the items analysed and the socio-demographic variables; and (c) bivariate correlations to establish the presence or absence of significant relations between the variables analysed. Parametric or non-parametric tests were used depending on the number of dependent and independent variables and on the assessment of the data series’ normality, randomness, and homoscedasticity (Table A1).

The qualitative phase used the programme NVivo 12 to determine the frequencies of occurrence of the words through a classic content analysis based on the criteria of Cabanillas-García, Luengo-González and Carvalho [41] and Cabanillas-García et al. [42]. The following steps were taken: (a) selection of the units to be analysed, parting from each of the selected units’ inclusion and exclusion criteria; (b) data reduction. The information was divided into units of grammatical content (paragraphs and sentences), after which the elements were identified and classified using a newly created codebook. This codebook was constructed using a mix of methods. One was inductive and consisted of creating categories based on the analysis of the collected material without taking into account any pre-established categories. The second was deductive and, as opposed to the previous one, used previously established categories into which the units were adapted. Thus, each unit initially received two codes from the research group that were reviewed until all researchers were satisfied both individually and as a group with the codes chosen. (c) assignation of values and grouping. The different graphic resources offered by NVivo were used to reveal the relations and discover the deep structure of the text, graphic representations, or visual images, of the relations between concepts and matrices, or double-entry tables, in whose cells a short verbal note is placed according to the aspects specified by columns and rows; (d) obtaining results and verifying conclusions. This implies the use of metaphors and analogies, as well as cartoons showing narrative fragments and interpretations by the research team. Finally, the results of the first and second stages are compared and interpreted by way of a SWOT analysis (strengths, weaknesses, opportunities, and threats).

The methods used to choose the sample subjects were convenience sampling and selection of key participants based on pertinence, adequacy, convenience, opportunity, and availability to participate in the research project. The result was a sample of 149 guidance counsellors. Table 1 shows the socio-demographic characteristics of the participants.

The instrument used is a semi-structured questionnaire comprising a total of 10 questions selected in the following manner. Four quantitative questions were obtained from the validated CCPES-II questionnaire on the self-perception of teaching competences, with the selected items being related to conflict management. Six qualitative questions were elaborated by the focus groups. These were designed to collect wider information on aspects related to intervention and support offered and aimed to provide a more holistic and real picture.

## 3. Results

### 3.1. Descriptive QUAN Data Analysis

This section presents the descriptive results of the four items (Table 2). Half of the guidance counsellors agree or totally agree (52.4%) with the need for more training in conflict management in the classroom or school. Likewise, almost two-thirds of the guidance counsellors (63.8%) agree or totally agree that conflict management is part of their duties as a guidance counsellor. However, almost half (40.3%) disagree or strongly disagree with the statement that in their profession, it is difficult to anticipate and prevent conflicts. Furthermore, the same proportion (40.3%) agrees or totally agrees that it is difficult to manage relations with students’ families.

### 3.2. Inferential QUAN Data Analysis

Here, the statistically significant differences are determined for each of the four items depending on the nominal socio-demographic variables (type of centre, province, current occupation, and sex) and ordinal variables (experience and age). A 95% confidence interval and 5% margin of error were used. Table 3 shows the *p*-values for each of the research items by nominal-variable group.

Next, Table 4 presents the p-value results and correlation coefficients (CC) for each correlation between the items, experience, and age.

Finally, Table 5 presents the correlations between the items themselves. A significant positive correlation can be seen between the need for training for guidance counsellors and the perception that in their profession it is difficult to anticipate and prevent conflicts (*p* = 0.000; CC = 0.314) and the consideration that the relationship with students’ families is a difficult area to manage (*p* = 0.001; CC = 0.273).

This indicates a strong link between guidance-counsellor training and conflict management with students, conflict management at the centre, and the relationship with families.

### 3.3. Qualitative Content Analysis

The content analysis was based on the following six analytical categories:

Category 1: Intervention measures to deal with conflicts. This category shows the main intervention measures or techniques used by guidance counsellors to resolve conflicts occurring in the education centre.

Category 2: Main conflicts. This shows the conflicts that most frequently take place in the education centre.

Category 3: Areas in which to train for conflict resolution. This category presents the most important areas in which guidance counsellors must train in order to become better at resolving conflicts in the education centre.

Category 4: Support for addressing conflicts. This shows aspects needed to more efficiently address conflicts.

Category 5: The feeling—or lack thereof—of fulfilment as a teacher. This shows whether or not the guidance counsellor’s pedagogical activity leaves them feeling fulfilled. It also highlights the causes that make them feel unfulfilled.

Category 6: Involvement of faculty and the administration in the resolution of conflicts. This category shows guidance counsellors’ perceptions of the level of involvement on the part of the centre’s administrative team and faculty in the resolution of conflicts.

**Figure 2:**
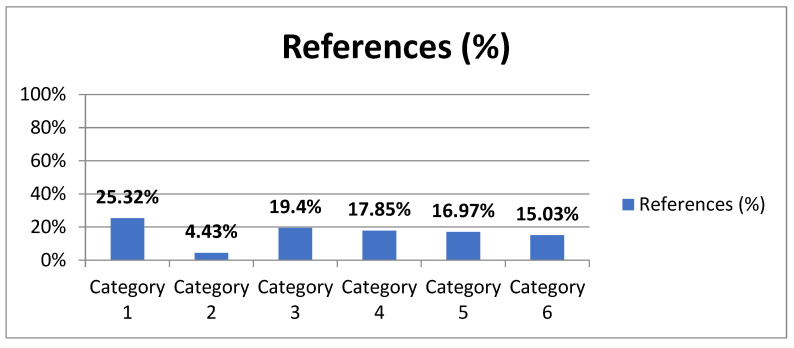
Reference frequency of analysis categories.

First of all, Figure 2 shows the reference frequency of each of the analysis categories. The categories with the strongest impact are categories 1 (23.32%) and 3 (19.4%). The category with the least repercussions is category 2 (4.43%).

The different categories will now be analysed in depth. Table 6 presents the intervention measures mentioned by guidance counsellors that are linked to category 1. The most prominent one is mediation (40 references), i.e., the intervention by a person or group in an argument between two parties with the aim of finding a solution, as pointed out by counsellors 73—"The most important thing for resolving conflicts is mediation among peers”—and 106—"Mediation between the parties involved is necessary”.

Also important is individual counselling and intervention with all parties involved (31 references), as indicated by counsellors 86—"I work in an office, so my sphere of influence is limited to one kid”—and 124—"I believe the most efficient approach is to work individually with the student”.

Other notable measures are dialogue and assertive communication (25 references), counselling and intervention with families, group counselling and intervention (22 references), and following the norms and protocols established by the centre or institutions (19 references).

Regarding the main conflicts linked to category 2 that guidance counsellors have to deal with, Table 7 shows that the most frequently mentioned ones are to do with people’s behaviour. On the one hand, counsellors mention behaviour problems (11 references). In the words of counsellor 23: “I have encountered behaviour problems in students such as disobedience and, on certain occasions, aggressiveness”. Counsellor 45 stated, “On certain occasions, students show certain behaviour problems”. Also prominently featured is conduct disorders (11 references). Counsellor 108 had this to say: “Conflicts arise among students due to some conduct disorders, such as hyperactivity”, to which counsellor 147 added: “I have experienced serious conflicts due to conduct disorders in students”.

Also important is disruption (10 references), i.e., behaviour that alters discipline or harmonious school relations, hindering or rendering impossible the teaching/learning process. Counsellor 58 said: “I have come across disruptive behaviours, such as constantly challenging me during class”, and counsellor 88 recounted: “The flow of classes is often interrupted with disruptive behaviours such as talking when I am explaining something or standing up at inappropriate moments”.

Finally, indiscipline and other types of behaviour related to the centres are also mentioned relatively often (both have nine references).

Regarding the subjects related to conflict resolution that counsellors would like to be trained in, subjects that fall into category 3, Table 8 highlights intervention and conflict-resolution strategies (34 references), about which counsellor 12 said: “Any related with conflict management; it’s always good to acquire new tools”, and counsellor 104: “Tools for working with teachers and students on resolving conflicts inside and outside the centre”.

Guidance counsellors also spoke about wanting training in mediation techniques, with counsellor 86 saying: “We should receive training in mediation techniques for use in the school and with families,” and counsellor 116: “We also need mediation training for students for conflict resolution among peers”.

Other subjects related to conflict resolution that guidance counsellors wanted to be trained in include prevention programmes (16 references), such as the TEI programme (peer tutoring) or the KIVA project (a programme against school bullying developed by the Finnish ministry of education); training in managing relations with families (15 references); emotional education (14 references); and techniques and resources for improving relations and atmosphere in the classroom (10 references). 

The need for help dealing with conflicts, covered in category 4, is shown in Table 9. Guidance counsellors find it necessary for the figure of a professional or team specialised in harmonious school relations to be included in educational centres (24 references), as stated by counsellors 73—"Creating a commission for the promotion of harmonious relations in which all involved sectors are represented”—and 85—"Personnel specialised in harmonious school relations in the centres”.

Guidance counsellors also emphasise the need for more time (20 references), as stated by counsellor 45: “Work strain also generates a lack of time, which causes a lack of proper attention to conflict situations” and counsellor 100: “We have insufficient time to devote to this type of intervention”. Counsellors also expressed their opinion that educational teams needed to be more involved (19 references). Counsellor 56 said: “Greater involvement from teachers”, and counsellor 93 stated: “commitment on the part of teachers and the centre’s management”.

Guidance counsellors also said they need more training (18 references) and support from families (15 references) to work from home on cases of conflict resolution they started at work, in a calmer atmosphere, one of cooperation and coordination with teachers (14 references).

As to whether guidance counsellors feel fulfilled in their jobs, the subject of category 5, only 16% of references indicate job satisfaction, while 86% report deficiencies and not feeling completely fulfilled. Table 10 presents the causes of guidance counsellors’ feelings of non-fulfilment, the most prominent being the need for more recognition, respect, and appreciation of one’s work (44 references), as stated by counsellors 12—“More recognition of the work and functions a pedagogue performs in any area of and moment in a person’s life. Not limiting it to education”—and 26—“More recognition of the activity we realise as pedagogues”.

Other major reasons why guidance counsellors say they do not feel fulfilled are needing more time (16 references), a lack of training (15 references), more support in the workplace (15 references), and more collaboration and cooperation between colleagues (15 references).

Regarding the involvement of teaching staff and school administrations in the resolution of conflicts, a subject covered by category 6, Table 11 shows that a majority of guidance counsellors feel these two collectives are committed (51 references), as expressed by counsellor 46—"In the centres where I have collaborated until now I do feel that both collectives were committed to helping or supporting students and to taking one common approach to resolving the conflict or difficulty”—and counsellor 101: “Yes, in my centre all teachers work and make efforts to improve”. Those that do not feel these two collectives are committed are a minority (38 references).

**Figure 3:**
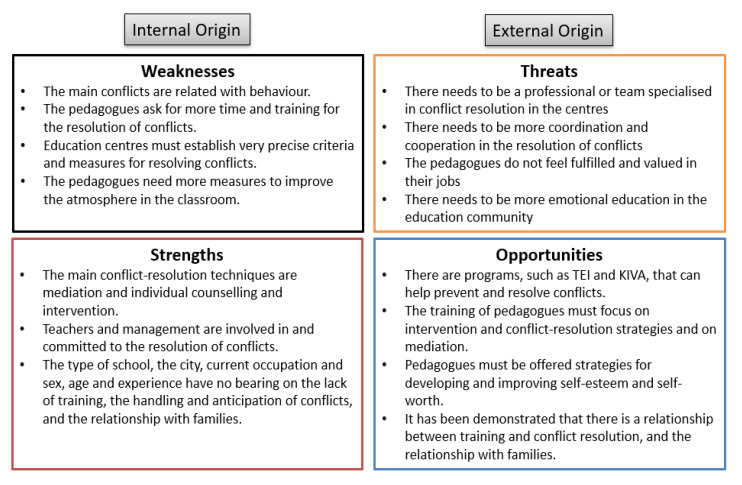
QUAN-QUAL integrated SWOT analysis.

To end, the following SWOT analysis brings together the QUAN and QUAL results to show the main internal and external conditions that were observed (Figure 3).

## 4. Discussion

Half of the guidance counsellors that participated in the study agreed on the need for more training in conflict management in the classroom or in the school. These results are in alignment with the results from several recent studies [33,35,36] that show there may be an important relationship between training in competences and strategies for conflict resolution, rapid intervention, and even the prevention of conflicts. Two thirds of the participating guidance counsellors agree that managing conflicts in their centres is part of their duties as a counsellor and consider that conflicts can be prevented. However, the same percentage indicates that some conflicts are difficult to anticipate and prevent because the variables that cause them do not lie exclusively in the educational centres [25,28,29]. Some authors argue that, even though conflicts have multiple causes, serious forms of conflict have indicators that help to intervene early [25,26,27,28,29,30,31,32]. On the other hand, almost half of the guidance counsellors say that the conflicts that most worry them are those that concern relations within the centres themselves—with their colleagues and with families—and not so much with students. The studies conducted by different pedagogical associations in other countries find and denounce the same thing: there are no contents related to conflict resolution in the training curricula of both teachers and, especially, guidance counsellors [34]. This study also points out the need for real training programmes in classroom management for fledgling guidance counsellors because this can provide multiple benefits down the road in their professional practise as well as help to reduce the stressful impact of having to deal with the personal and even family-related problems of students and their families [35,36]. Curiously, no studies were found that measure stress levels or burnout syndrome in guidance counsellors working in school centres, studies that do exist for teachers.

This study found the most important types of conflict facing guidance counsellors to be disruption, behaviour problems, conduct disorders, and emotional problems. This conclusion agrees with current post-pandemic research, which describes children and adolescents’ unmet emotional needs, their loneliness, anxiety, and depression as the most important consequences of the pandemic, together with the uncontrolled use, or rather abuse, of the new technologies [11,15,43]. Violence and bullying, however, appear to be less present in the situations described by this study’s participants. This does not coincide with other studies for which violence is the biggest problem currently facing schools [27,28,29,30]. It does, however, coincide with other studies that maintain that violence is preceded by a variety of conflicts, which makes it possible to prevent it [29,31,32].

Regarding intervention measures for resolving conflicts, the results obtained in this study show the most successful ones to be mediation (both individually and at the group level) and individualised attention for students. Likewise, the most requested types of continuous training are communication strategies, social skills such as assertiveness, strategies for managing and attending families, and knowing the different intervention protocols for each type of conflict [33]. There is also a demand for concrete training programmes for conflict resolution, such as the TEI programme (peer tutoring) [44] or the KIVA project (a programme against school bullying developed by the Finnish ministry of education) [45], and for training in the management of relations with families and for emotional education.

The results also expose the need for strengthening psycho-pedagogical teams with professionals specialised in promoting harmonious school relations. This demand follows the current trend among schools that have, in recent years, created commissions to promote harmonious school relations and included the figure of the school coexistence and equality manager in their staff.

Finally, in order to feel fulfilled in their professional duties, guidance counsellors demand more time to perform the functions inherent to attending students, as well as more recognition of their work, but only from teachers because most counsellors say they already feel supported by school administrators.

## 5. Conclusions

The COVID-19 pandemic has considerably increased mental-health problems among children and adolescents. Anxiety, stress, despair, fear, etc., have all increased because of the confinement and fear of infection. These problems, which have sometimes resulted in behaviour problems, have arrived in schools, where guidance counsellors have been the professionals tasked with tackling and providing support in these situations. To perform this task, they need specific competences—some of them socio-emotional—in which they have usually not been trained. The results of this and other similar studies confirm the need for specific training in socio-emotional competences, intervention strategies, family and classroom management, and techniques and instruments for prevention and mediation. Also needed are more specialised staff in schools to address mental-health problems in children and adolescents, more time to address these problems, more training, firm support from families, and recognition from colleagues, families, and society of professionals’ work and dedication. Future research should further diagnose and analyse guidance counsellors’ and teachers’ training deficiencies regarding socio-emotional competences so as to implement continuous-training programmes that help them respond to the problem of conflicts in schools. Finally, this study shows that the professional recognition of guidance counsellors should be compensated with more staffing in the guidance unit, better working conditions depending on the results obtained by each guidance team, and more visibility for their “personal brand” of good practises in guidance.

The limitations of this research revolve around the sample. Future research should, on the one hand, increase the number of school counsellors analysed. In addition, the percentage of school counsellors should be balanced by cities in order to be able to establish comparisons between the different Spanish localities. Finally, it would also be interesting to adjust the number of school counsellors according to sex so that the socioemotional competencies of the counsellors could also be analysed according to this variable.

## Figures and Tables

**Table 1:** Socio-demographic characteristics of the sample.

Variable	Mean ± SD/Frequency (Percentage)
Type of centre	
Public	46 (30.9%)
Concerted	92 (61.7%)
Private	11 (7.4%)
Experience	10.82 ± 7.69
City	
Castellón	12 (8.1%)
Valencia	58 (39.9%)
Alcoy	2 (1.3%)
Granada	7 (4.7%)
Zaragoza	3 (2%)
Málaga	4 (2.7%)
Sevilla	5 (3.4%)
Alicante	25 (16.8%)
Madrid	9 (6%)
Cádiz	3 (2%)
Las Palmas de Gran Canaria	3 (2%)
Cuenca	4 (2.7%)
Palencia	2 (1.3%)
Melilla	1 (0.7%)
Teruel	1 (0.7%)
Córdoba	1 (0.7%)
Murcia	4 (2.7%)
Mallorca	1 (0.7%)
Toledo	2 (1.3%)
Ceuta	1 (0.7%)
Melilla	1 (0.7%)
Current occupation	
School counsellor	131 (87.9%)
External	18 (12.1%)
Age	38.65 ± 9.27
Sex	
Men	22 (14.8%)
Woman	127 (85.2%)

Data shown as mean ± standard deviation (SD) for quantitative variables and absolute frequency (percentage) for ordinal variables.

**Table 2:** Descriptive results of the scale variables.

Variable	Strongly Disagree	In Disagreement	Indifferent	In Agreement	Totally Agree
I consider that I need training to manage conflicts in the classroom or at the centre	6 (4%)	22 (28.9%)	43 (14.8%)	50 (33.6%)	28 (18.8%)
The management of conflicts in the centre is part of my duties as a counsellor	10 (6.7%)	14 (9.4%)	30 (20.1%)	52 (34.9%)	43 (28.9%)
In my profession it is difficult to anticipate conflicts and prevent them	8 (5.4%)	52 (34.9%)	64 (43%)	19 (12.8%)	6 (4%)
The relationship with the families of the students is a difficult area to manage	11 (7.4%)	33 (22.1%)	45 (30.2%)	52 (34.9%)	8 (5.4%)

Absolute frequency (percentage) for scale variables.

**Table 3:** Results based on the research items and groups of nominal variables.

Variable	Type of Centre (H de Kruskal–Wallis)	City (H de Kruskal–Wallis)	Current Occupation (U de Mann–Whitney)	Sex (U de Mann–Whitney)
Item 1	*p* = 0.841	*p* = 0.707	*p* = 0.384	*p* = 0.357
Item 2	*p* = 0.337	*p* = 0.365	*p* = 0.619	*p* = 0.247
Item 3	*p* = 0.102	*p* = 0.493	*p* = 0.983	*p* = 0.470
Item 4	*p* = 0.317	*p* = 0.890	*p* = 0.673	*p* = 0.079

*p*-values between groups.

**Table 4:** Correlation between research items, experience, and age.

Variable	Experience (Pearson’s Correlation)	Age (Pearson’s Correlation)
Item 1	CC = −0.142	CC = −0.056
	*p* = 0.085	*p* = 0.500
Item 2	CC = 0.108	CC = 0.062
	*p* = 0.191	*p* = 0.454
Item 3	CC = 0.011	CC = 0.089
	*p* = 0.897	*p* = 0.279
Item 4	CC = −0.020	CC = −0.043
	*p* = 0.813	*p* = 0.601

*p*-values and CC.

**Table 5:** Correlations between items.

Variable	Item 1	Item 2	Item 3	Item 4
Item 1	X	CC = 0.062	CC = 0.314	CC = 0.273
		*p* = 0.449	*p* = 0.000 **	*p* = 0.001 **
Item 2	CC = 0.062	X	CC = −0.038	CC = 0.129
	*p* = 0.449		*p* = 0.646	*p* = 0.119
Item 3	CC = 0.314	CC = −0.038	X	CC = 0.122
	*p* = 0.000 **	*p* = 0.646		*p* = 0.139
Item 4	CC = 0.273	CC = 0.129	CC = 0.122	X
	*p* = 0.001 **	*p* = 0.119	*p* = 0.139	

*p*-values and CC (** Correlation is significant at the 0.01 bilateral level).

**Table 6:** Analysis of category 1. Intervention measures to deal with conflicts.

Subcategory	References (n)
Mediation	40
Individual counselling and intervention	31
Dialogue and assertive communication	25
Counselling and intervention with families	22
Group counselling and intervention	22
Following norms and protocols	19
Behaviour modification	14
Social skills and emotional intelligence programme	10
Punishment or penalty	10
Coordination and guidance with teachers	10
Counselling and intervention at the level of the centre	6
Coordination with other areas	5
Knowing and analysing the conflict	5
Tutoring	4
Group dynamics	4
Follow-up	4
Prevention	4
Reprimand	3
Positive reinforcements	3
Reflection	3
Justice	2
Commitment form	2
Reflection sheets	2
Sociograms	2
Strategies for self-control	2
Authority	1
Motivation	1
Observation	1
Looking for solutions	1
Considers it is not included in their duties	1
Referring to the guidance department	1
Looking for an agreement between the affected parties	1

Number of references in the analysed text assigned to each subcategory.

**Table 7:** Analysis of category 2. Main conflicts.

Subcategory	References (n)
Behaviour problems	11
Conduct disorders	11
Disruption	10
Indiscipline	9
Other related to the centres	9
Bullying	3
Violence between students	2
Cyberbullying	1

Number of references in the analysed text assigned to each subcategory.

**Table 8:** Analysis of category 3. Areas in which to train for conflict resolution.

Subcategory	References (n)
Intervention and conflict-resolution strategies for teachers	34
Mediation	29
Strategies against inappropriate behaviour among students	18
Prevention programs	16
Managing relations with families	15
Emotional education	14
Improving relations and atmosphere in the classroom	10
New tendencies and perspectives	9
Cyberbullying	8
Techniques and strategies for students with disabilities	6
Bullying	4
Improving self-control	4
Techniques for maintaining discipline	4
Social skills	4
New technologies	3
Presenting real cases	2
None	2
Educational coaching	2
Motivation strategies	2
Addiction	2
Strategies for permanent, not temporary solutions	2
Techniques that do not use punishments or prizes	2
On cultural management	1
Improving self-esteem	1
Sex education	1
Legislation	1
Practical intervention in students	1
Stress management	1
Brief and systematic therapy	1
Individualised attention for students	1

Number of references in the analysed text assigned to each subcategory.

**Table 9:** Analysis of category 4. Support for addressing conflicts.

Subcategory	References (n)
Figure of a professional or team specialised in harmonious school relations	24
Time	20
Involvement of the educational team	19
Training	18
Support from families	15
Cooperation and coordination with teachers	14
More guidance counsellors	11
Teachers or support personnel	11
More resources	10
Real measures and not so much bureaucracy	6
Following the intervention protocol	5
More pedagogues	4
More social educators	4
More interventions by social services	4
Coexistence classrooms	4
Swift response	3
More prevention plans and strategies	3
Information	2
More psychologists	2
Awareness-raising campaigns	2
Figure of a mediator (teacher or student)	1
Guidance team working more closely together	1
Social integrator	1

Number of references in the analysed text assigned to each subcategory.

**Table 10:** Analysis of category 5. The feeling—or lack thereof—of fulfilment as a teacher.

Subcategory	References (n)
More recognition, respect, and appreciation of one’s work	44
More time	16
Lack of training	15
More support in the workplace	15
More collaboration and cooperation between colleagues	15
Being able to better perform my professional functions	6
Feeling less pressure in my work	6
Permanent full-time contract instead of an internship	4
Better salaries	4
We need more resources	4
More commitment from colleagues	3
More collaboration on the part of families	3
Working on emotional education	2
Hiring more professionals for the guidance team	2
Being able to see the result of my work in students	2
Less intrusion in the workplace	2
Continuing to improve as a teacher	1
Acquiring more experience	1
More work and contact with the classrooms	1
New challenges	1

Number of references in the analysed text assigned to each subcategory.

**Table 11:** Analysis of category 6. Involvement of faculty and the administration in the resolution of conflicts.

Subcategory	References (n)
I do feel both collectives are committed	51
I do not feel both collectives are committed	38
Sometimes I feel both collectives are committed	33
It depends on the person	9
I feel management is committed but teachers are not	8
Both collectives need to be more involved	7
It depends on the centre	3
There is only involvement in serious situations	3
Only at the beginning of the school year	1
It depends on age	1
It depends on training	1

Number of references in the analysed text assigned to each subcategory.

## Appendix A

The tests applied to contrast the distribution of the sample and the behaviour of the sample in the qualitative analyses are presented below.

**Table A1:** Tests applied to the qualitative items.

Variable	Normality (Kolmogorov–Smirnov)	Randomness (Rachas)	Homoscedasticity (Levene)	Test
I consider that I need training to manage conflicts in the classroom or at the centre	0.000	0.784		Non-parametric
The management of conflicts in the centre is part of my duties as a counsellor	0.000	0.980		Non-parametric
In my profession it is difficult to anticipate conflicts and prevent them	0.000	0.068		Non-parametric
The relationship with the families of the students is a difficult area to manage	0.000	0.690		Non-parametric
